# Supplementary material for: Evidence of inter-state coordination amongst state-backed information operations
Source: Sci Rep. 2023 May 12;13:7716. doi: 10.1038/s41598-023-34245-1 (PMC10182002; doi:10.1038/s41598-023-34245-1)
Supplement: Supplementary file 1 — Supplementary Information. [file 41598_2023_34245_MOESM1_ESM.pdf]

Supplementary Material

	Cuba	Venezuela	Russia	Iran
**Administrator**	2	0	0	0
**Influencer**	4	14	10	1
**Promoter**	15	4	2	1
**Broker**	5	2	0	0
**Follower**	30	42	42	327
**Total**	56	62	54	329

Table S1: Role analysis of Cuba-Venezuela and Russia-Iran coordination.

Coordination	Source	Category	Active	Count
Cuba-Venezuela	Profile	Instagram	Yes	2
		Facebook	Yes:2 No:3	5
		Blogspot	Yes	3
		Invalid		6
	Content	Twitter	Yes:4 No:29	33
		Facebook	Yes	1
		YouTube	Yes:15 No:4	19
		Blogspot	Yes	165
		News sites	Yes:305 No:21	326
		Radio sites	Yes	2
		Miscellaneous sites	Yes:1 No:8	9
		Social media content management	Yes	17
		Invalid		1,341
Russia-Iran	Profile	Instagram	No	2
		Facebook	No	1
		Telegram	Yes	1
		Miscellaneous sites	Yes	2
		Invalid		1
	Content	Instagram	No	1
		Twitter	No	3
		News sites	Yes:11 No:5	16
		Miscellaneous sites	Yes	1
		Invalid		14

Table S2: URLs contained in profile descriptions and shared content, within Cuba-Venezuela and Russia-Iran inter-state operations.

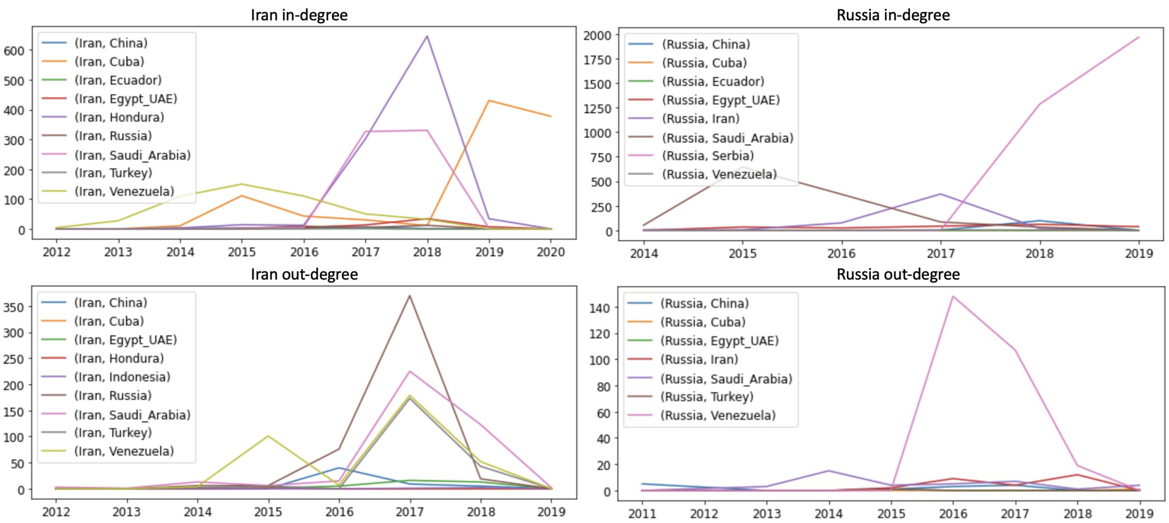

Figure S1: Dynamic in-/out-degree for Russia and Iran in global inter-state coordination.

**Country**	**Avg like**	**p**	**T**	**Avg retweet**	**p**	**T**	**Avg reply**	**p**	**T**
VE(Inter)	0.401	0.988	-0.015	0.440	0.193	1.301	0.128	0.402	-0.837
VE(External)	0.442			0.939			0.076		
CU(Inter)	0.539	0.838	0.205	0.366	0.087	1.712	0.125	**0.001****	-3.432
CU(External)	0.706			0.514			0.072		
CN(Inter)	0.035	**0.013***	2.473	0.058	**0.005****	2.818	0.351	**0.000*****	-4.006
CN(External)	0.463			0.295			0.221		
IR(Inter)	0.056	**0.032***	2.148	0.052	**0.021***	2.303	0.101	0.199	0.021
IR(External)	3.113			0.136			x		
RS(Inter)	0.000	**0.000*****	4.008	0.000	**0.000*****	3.844	0.000	**0.000*****	4.186
RS(External)	0.101			0.080			0.010		
RU(Inter)	0.007	**0.015***	2.426	0.000	**0.008****	2.638	0.018	0.079	1.755
RU(External)	27.240			11.578			0.578		
ID(Inter)	0.034	0.872	0.290	0.072	0.876	-0.156	0.559	**0.000*****	-27.193
ID(External)	0.090			0.040			0.174		
SA(Inter)	0.037	0.166	1.385	0.068	0.058	1.898	0.034	0.052	0.058
SA(External)	0.991			1.007			0.298		
EC(Inter)	0.020	0.460	0.739	0.002	0.221	1.224	0.000	0.386	0.867
EC(External)	0.030			0.027			0.039		
HN(Inter)	0.450	0.593	0.535	0.619	0.575	-0.561	0.030	0.126	0.532
HN(External)	0.610			0.440			0.193		
EU(Inter)	0.073	0.057	1.907	0.076	**0.001*****	3.258	0.036	**0.001*****	0.001
EU(External)	0.380			0.403			0.069		

Table S3: Comparison of inter-state and external engagement. p: p-value; T: T-score; *: $p < 0.05$ (significant), **: $p < 0.01$ (highly significant), ***: $p < 0.001$ (extremely significant).
